# Lactylation-related gene signatures identify glioma molecular subtypes with prognostic, immunological, and therapeutic implications

**DOI:** 10.3389/fonc.2025.1613423

**Published:** 2025-07-16

**Authors:** Yanliang Tang, Xiaoli Zhang, Xiaofei Tang, Ye Yuan, Wenwen Wang

**Affiliations:** Department of Neurology, Fuyang Hospital of Traditional Chinese Medicine, Hangzhou, Zhejiang, China

**Keywords:** glioblastoma, lactylation, subtype, prognosis, infiltration

## Abstract

**Introduction:**

Lactic acid is a by-product of energy metabolism and a signaling molecule that influences tumor progression by regulating immune cell function, angiogenesis, and epigenetic modifications.

**Methods:**

This study analyzed data from the TCGA database on gliomas to systematically elucidate the expression patterns, prognostic value, and functional regulatory networks of lactylation-related genes.

**Results:**

In this study, 17 lactylation-related prognostic genes were identified through the analysis of TCGA-GBM data. Using non- negative matrix factorization (NMF), two GBM subtypes based on lactylation- related genes (LRGs), termed GBM1 and GBM2, were identified. Survival analysis revealed that the overall survival (OS) of the GBM1 group was significantly lower than that of GBM2 group. Furthermore, notable differences were observed in the expression of key GBM-associated molecular markers between the two subtypes. Tumor microenvironment (TME) analysis demonstrated distinct immune landscapes and genomic characteristics between GBM1 and GBM2. The GBM1 group exhibited higher immune cell infiltration and immune function scores compared to GBM2. Drug sensitivity analysis further revealed differences in response to chemotherapy and targeted therapies between the two subtypes. In vitro data demonstrated that LCP1 knockdown suppressed cell proliferation and invasion, and promoted apoptosis in glioma cells.

**Conclusion:**

In conclusion, our study systematically uncovers the significant role of LRGs in GBM molecular subtyping, prognosis evaluation, and therapeutic guidance. These findings offer new insights and potential strategies for the personalized treatment of GBM.

## Introduction

Gliomas are the most common primary malignant tumors of the central nervous system, accounting for over 30% of all brain tumors. Among them, glioblastoma multiforme (GBM; WHO grade IV) has garnered significant attention due to its pronounced invasiveness, high recurrence rate, and extremely poor prognosis (1). Despite continuous advancements in multimodal therapies, including surgery, radiotherapy, and chemotherapy, the median survival of GBM patients remains under 15 months, with a 5-year survival rate of less than 5% (2). This grim situation underscores the urgent need to deeply elucidate the molecular mechanisms driving glioma malignancy. In recent years, the intersection of tumor metabolic reprogramming and epigenetic regulation has offered new perspectives for glioma biology and potential treatment strategies. A hallmark of gliomas is the Warburg effect, or aerobic glycolysis, which leads to the accumulation of substantial amounts of lactate in the tumor microenvironment (TME) (3). Traditionally regarded as a metabolic byproduct, lactate is now recognized as a critical signaling molecule that influences tumor progression by modulating immune cell function, regulating angiogenesis, and altering epigenetic landscapes (4, 5).

Posttranslational modifications (PTMs) are covalent alterations, either reversible or irreversible, that modify protein function and dynamics by adding chemical groups or cleaving peptide bonds, thereby regulating diverse biological processes (6). The types of PTMs include phosphorylation, ubiquitination, acetylation, methylation, glycosylation, sumoylation, and palmitoylation (7). PTMs have been validated in glioma progression, including ubiquitination, sumoylation, and acetylation (8–10). Lactylation is a newly discovered PTM first reported in 2019 (11), which involves the covalent attachment of lactyl groups that is derived from lactate to lysine residues on histones, directly regulating chromatin structure and transcriptional regulation (12). Lactylation plays a key role in macrophage polarization, immune regulation within tumors, and the cellular response to metabolic stress (13–16). For example, elevated lactate levels in the TME have been shown to induce histone H3K18 lactylation, thereby promoting the expression of pro-tumor genes such as PD-L1 and inhibiting anti-tumor immune responses (17, 18). Additionally, lactate can be reused by oxidative tumor cells as an energy substrate, facilitating tumor progression through a “metabolic symbiosis” model (19). Collectively, lactate is not merely a metabolic by product, but also a core regulatory factor of the malignancy and a potential therapeutic target. With the rapid advancement of high-throughput sequencing technologies, public databases such as TCGA, CGGA, and GTEx have become valuable resources for exploring the molecular heterogeneity of gliomas (20, 21). Prognostic models based on machine learning, including LASSO-Cox regression, have been successfully applied to identify metabolism-related genes in gliomas (22), while WGCNA has proven effective in detecting hub genes associated with lactylation modifications (23). Moreover, integrative analyses of epigenomics (e.g., ChIP-seq, ATAC-seq) and metabolomics offer the potential to uncover how lactylation influences chromatin accessibility and transcription factor activity (24, 25). Lactylation has been revealed to be involved in GBM development, prognosis and treatment (26). One group used an integrated analysis of transcriptome and scRNA sequencing, and identified prognostic genes related to histone lactylation in GBM (27). However, a comprehensive and systematic analysis of the regulatory network underlying lactylation modifications is still lacking. To date, no molecular classification system for gliomas based on LRGs has been established.

In this study, we systematically analyzed glioma data from the TCGA database to elucidate the expression patterns, prognostic value, and functional regulatory networks of LRGs. First, we screened differentially expressed LRGs (such as EEF1A1, S100A4, RPL5) and established a molecular subtyping system based on these genes. This classification stratified glioma patients into distinct molecular subtypes, allowing for prognostic assessment through survival analysis. subsequently, we utilized WGCNA and Gene Set Enrichment Analysis (GSEA) to reveal the biological pathways associated with these gene sets. Furthermore, we conducted protein-protein interaction (PPI) networks to predict potential downstream targets and interaction partners of lactylation regulators. This study is the first to systematically define molecular subtypes of GBM based on LRGs, thereby shedding light on the underlying molecular heterogeneity. Our findings may provide valuable insights into prognostic prediction and pave the way for personalized therapeutic strategies in GBM.

## Materials and methods

### Collection and processing of transcriptomic data

RNA sequencing (RNA-seq) data for GBM, along with corresponding clinical information, were downloaded from the Cancer Genome Atlas (TCGA) database (https://www.cancer.gov/about-nci/organization/ccg/research/structural-genomics/tcga). After filtering, 168 GBM samples with available survival and clinical data, as well as 5 normal brain tissue samples, were selected for analysis. Expression data were converted into transcripts per million (TPM) format and log2-transformed for subsequent analysis. Somatic mutation data were downloaded separately from the TCGA dataset. The LRGs were sourced from previous published studies (28). A PPI network of the LRGs was constructed using the STRING database (https://string-db.org/cgi/input.pl).

### Collection and processing of single-cell transcriptomics data

Single-cell RNA-seq data were obtained from the GEO database GSE271618, which comprises approximately 25,000 cells in total. Data analysis was conducted using R and the Seurat package. Quality control was performed by filtering cells based on mitochondrial and ribosomal gene content, setting gene expression thresholds between 200–1000 for mitochondrial and 200–2000 for ribosomal genes. Key Seurat functions used included NormalizeData, FindVariableFeatures, and ScaleData for normalization, identification of highly variable genes, and scaling, respectively. Principal component analysis (PCA) was performed using the “RunPCA” function. Batch effects across samples were corrected using the Harmony package, and dimensionality reduction and clustering were performed using uniform manifold approximation and projection (UMAP). Cell type annotation was based on established marker genes.

### Identification of differentially expressed and prognostic genes

Differential expression analysis between GBM patients and healthy controls were performed using the limma R package (version 3.60.6). Significantly differentially expressed genes (DEGs) were defined using the thresholds: adjusted P < 0.05 and |log2 fold change| > 1. Visualization of DEGs was conducted using the R packages pheatmap (version 1.0.12), dplyr (version 1.1.4), ggplot2 (version 3.5.1), and ggrepel (version 0.9.6).

By intersecting the DEGs with LRGs, 100 LRGs in GBM were identified. Univariate Cox regression analysis was then performed on these 100 genes with a threshold of p < 0.05, resulting in the identification of 17 prognostically relevant genes. A Venn diagram was generated using the VennDiagram package (version 1.7.3). A forest plot displaying hazard ratios (HRs) and p-value for the 17 genes was created using the survival R package (version 3.7.0).

### Identification of molecular subtypes of GBMs through consensus clustering

To classify GBM molecular subtypes, consensus clustering was performed using the 17 identified prognostic LRGs within the TCGA-GBM cohort. Non-negative matrix factorization (NMF) was applied via the NMF R package. The optimal number of clusters was evaluated for k values ranging from 2 to 5, with 1,000 iterations to ensure result stability. Heatmaps were generated using the pheatmap package to visualize clustering outcomes. PCA was used to determine whether the identified subtypes could effectively distinguish GBM samples. Survival differences between subtypes were evaluated using the survival and survminer packages to investigate the relationship between molecular subtypes and OS.

### Assessment of immune cell infiltration

TME characteristics were calculated for each GBM sample based on the gene expression patterns using the ESTIMATE, including stromal score, immune score and estimate score. To quantify immune cell infiltration, single-sample gene set enrichment analysis (ssGSEA) was performed using transcriptomic data and immune-relate gene sets. The Wilcoxon rank-sum test was employed to compare the immune cell infiltration levels between the two identified GBM subtypes.

### Drug sensitivity analysis

Drug sensitivity analysis was conducted using the pRRophetic R package (version 0.5), which builds prognostic models from gene expression profiles of cancer cell lines to estimate the drug response in patient samples. The half-maximal inhibitory concentration (IC50) values were calculated for selected chemotherapeutic and targeted drugs. Boxplots were used to visualize and compare differences in drug sensitivity (IC50 values) between the GBM subtypes.

### Weighted gene co-expression network analysis

WGCNA was performed on the TCGA-GBM dataset using the WGCNA package (version 1.73). The top 25% of genes with the highest variance across samples were selected for analysis. Pearson correlation coefficients were calculated to construct an adjacency matrix, with a soft-thresholding power of 5 applied to enhance network scale-freeness. The adjacency matrix was then converted into a Topological Overlap Matrix (TOM) and gene modules were identified using a dynamic tree-cutting algorithm. Within the identified modules, genes most strongly correlated with LRGs were defined as feature genes related to lactylation.

### Gene set enrichment analysis

Reference gene sets c5.go.v2024.1.Hs.symbols.gmt (GO) and c2.cp.kegg_medicus.v2024.1.Hs.symbols.gmt (KEGG) were downloaded from the Molecular Signatures Database (MSigDB) v4.0. GSEA was performed to assess pathway activity differences between the two molecular subtypes. DEGs between subtypes were identified using the limma package (version 3.60.6). Subsequently, GO and KEGG were conducted to describe the functional roles of the DEGs.

### Cell culture

Human glioma cell lines U251 and LN229 were bought from the Cell bank of the Chinese Academy of Science (Shanghai, China). Cells were maintained in RPMI-1640 medium supplemented with 10% fetal bovine serum at 37°C in a humidified atmosphere containing 5% CO_2_.

### Transfection

Lentivirus shRNAs targeting LCP1 (shRNA#1: GCG GAC ATT TAG GAA CTG GAT; shRNA#2: CCT GGG TAT AGA GTA CGA GAA) and a negative control shRNA were purchased from GenePharma (Shanghai, China). Transfections were performed using Lipofectamine 3000 Reagent (Invitrogen, Carlsbad, USA) according to the manufacturer’s protocol. Transfection efficiency was assessed by Western blotting.

### Western blotting

Total protein was extracted using RIPA lysis buffer and separated by SDS-PAGE, then transferred onto a PVDF membrane. Membranes were blocked with 5% skim milk at room temperature for 1 hour, followed by incubation with primary antibodies and then the appropriate secondary antibodies. The primary antibodies used were anti-LCP1 (1:1000, #5350, Cell Signaling Technology, MA, USA) and anti-GAPDH antibodies (1:5000, #2118, CST, MA, USA).

### CCK-8 assay

Cell viability was measured using the Cell Counting Kit-8 (CCK-8) assay. Glioma cells transfected with shLCP1 were seeded into plates and incubated for various time pints. Subsequently, cells were incubated with 10 µL of CCK-8 solution for 2 hours. The OD value at 450 nm was assessed using a microplate reader.

### EdU assay

Cell proliferation was evaluated using an EdU kit (Beyotime, China). Transfected cells were seeded into 24-well plates and cultured for 72 hours, followed by incubation with EdU solution for 4 hours. Cells were then fixed with 4% paraformaldehyde and treated with Click reaction mixture for half hour. Hoechst 33342 was used for nuclear staining. Images were acquired using a fluorescence microscope, and the proportion of EdU-positive cells were quantified using ImageJ software.

### Apoptosis assay

Apoptosis was analyzed using an Annexin V-FITC/PI apoptosis detection kit. Following shLCP1 transfection, glioma cells were harvested, washed, and resuspended in 500 μL of 1×binding buffer. Then, 5 µL of Annexin V-FITC and 10 μL of propidium iodide (PI) were added. Apoptotic cells were detected using flow cytometry.

### Transwell invasion assay

Cell invasive capacity was measured using Transwell chambers (Corning, USA) pre-coated with Matrigel. The lower chambers were filled with medium containing 10% fetal bovine serum. Transfected cells suspended in serum-free medium were added to the upper chamber. After 24 hours of incubation, non-invading cells were removed by a cotton swab. Invading cells on the lower surface of the membrane were fixed with 4% polyformaldehyde, stained with crystal violet, and imaged. The number of invaded cells was counted under a macroscope.

### Statistical analysis

All statistical analyses were performed using R software (version 4.4.1) and relevant R packages. For Wilcoxon rank-sum tests, chi-square test, Kaplan–Meier survival analysis, and t-test, a p-value < 0.05 was considered statistically significant. For GO, KEGG, and GSEA analyses, a false discovery rate (FDR) < 0.05 was used to determine statistical significance.

## Results

### Identification of lactylation-related prognostic genes in GBM

**Figure 1:**
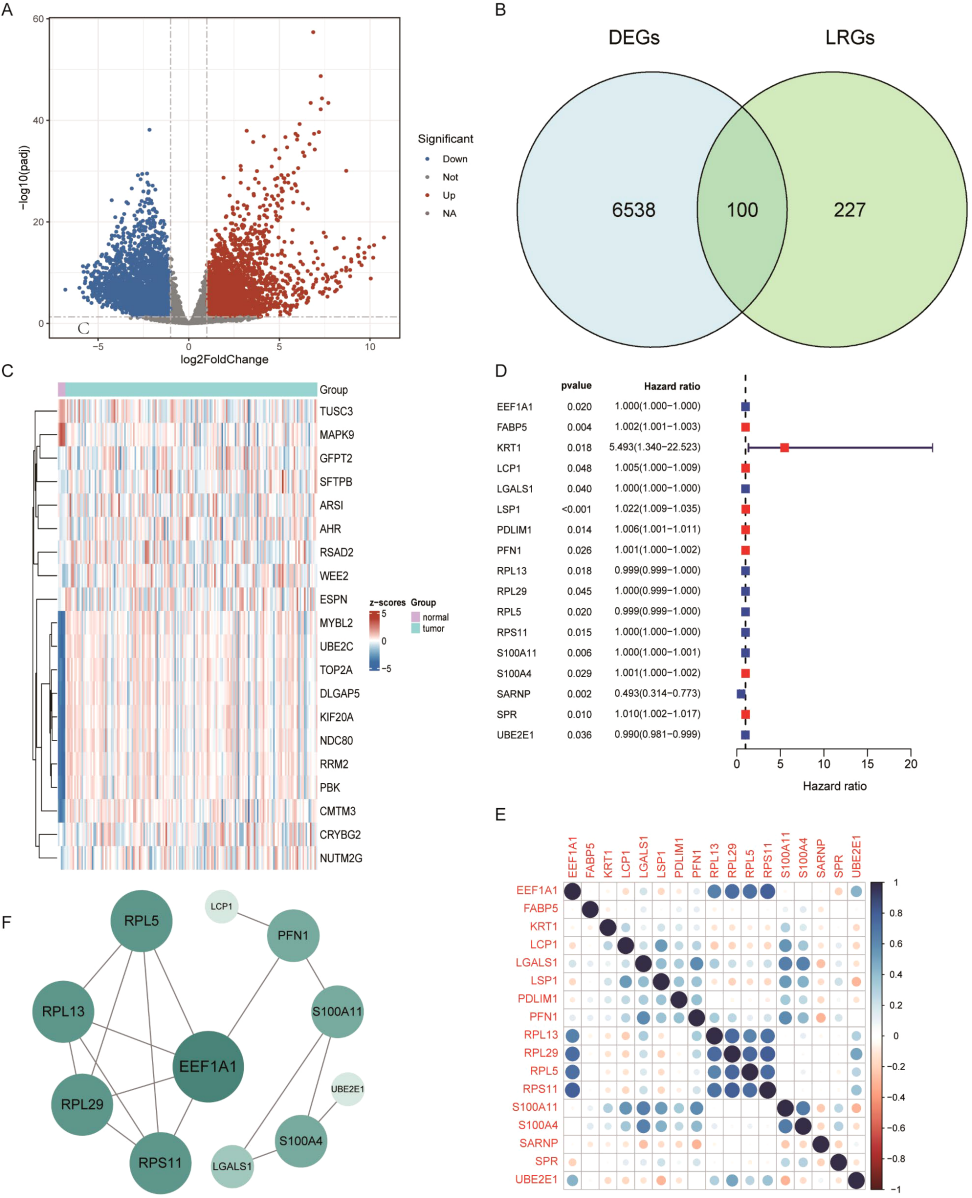
Differential expression analysis of lactylation-related genes in GBM. **(A)** Volcano plot of differentially expressed genes (DEGs) in GBM. **(B)** Venn diagram showing the overlap between glioblastoma DEGs and lactylation-related genes. **(C)** Heatmap displaying the top 40 lactylation-related DEGs. **(D)** Forest plot of prognostic lactylation-related DEGs, based on univariate Cox regression. **(E)** Correlation analysis among intersecting genes. **(F)** Protein–protein interaction (PPI) network illustrating known and predicted interactions among prognostic lactylation-related DEGs.

First, mRNA expression data from 168 GBM tumor tissues and 5 normal brain tissues were obtained from the TCGA database, along with corresponding clinical information. Differential expression analysis was performed, a volcano plot displayed significantly upregulated and downregulated genes among all samples (**Figure 1A**). The differential genes of P <0.05 (**Supplementary Figure 1A**). By intersecting 6,638 DEGs with 327 previously reported LRGs, a total of 100 GBM-associated LRGs were discovered (**Figure 1B**). The top 20 differentially expressed LRGs were visualized in a heatmap (**Figure 1C**). Univariate Cox regression analysis was then conducted on the 100 candidate genes, resulting in the identification of 17 prognostic LRGs: EEF1A1, FABP5, KRT1, LCP1, LGALS1, LSP1, PDLIM1, PFN1, RPL13, RPL29, RPL5, RPS11, S100A11, S100A4, SARNP, SPR, and UBE2E1. These genes were associated with varying hazard ratios, as shown in the forest plot (**Figure 1D**). A gene correlation heatmap highlighted strong positive correlations between several genes at the transcriptomic level, including EEF1A1 with RPL13, RPL29, RPL5, and RPS11; LCP1 with LSO1 and S100A11; LGALS1 with PFN1, S100A11, and S100A4; PFN1 with LGALS1 and S100A11 (**Figure 1E**; **Supplementary Figure 1B**). To explore protein-level interactions, a PPI network was constructed using the STRING database. The network revealed extensive interconnectivity among proteins encoded by these prognostic genes, including both known and predicted interactions (**Figure 1F**).

### Identification of novel GBM subtypes via unsupervised learning

**Figure 2:**
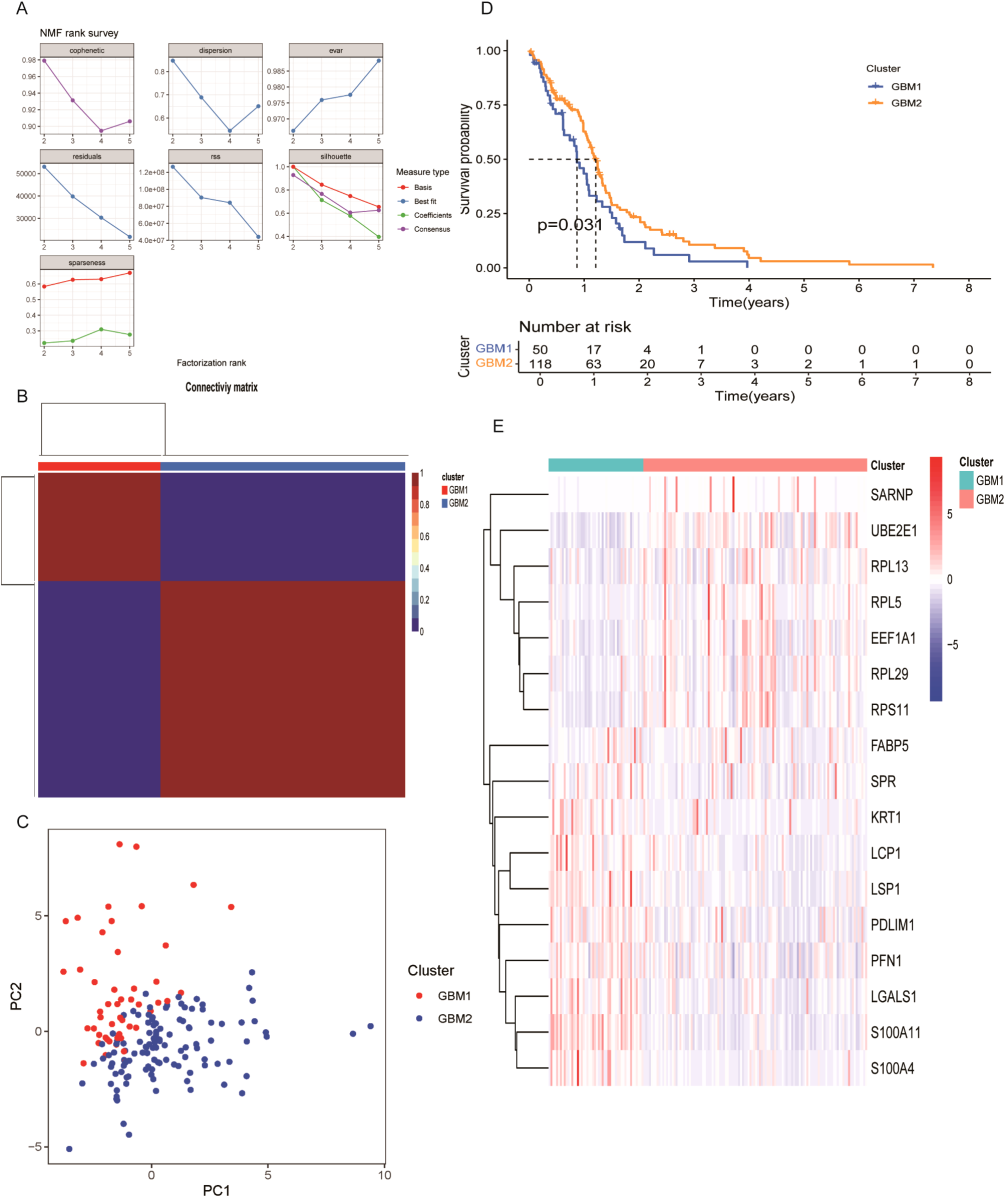
Molecular subtype classification of GBM based on lactylation-related DEGs. **(A)** Consensus clustering identified two distinct subtypes (k = 2). **(B)** Consensus matrix heatmap and cumulative distribution function (CDF) plot illustrating clustering stability. **(C)** Principal component analysis (PCA) showing clear separation between GBM1 and GBM2 subtypes. **(D)** Kaplan–Meier survival curves comparing overall survival between the two subtypes. **(E)** Expression patterns of 17 lactylation-related DEGs across GBM1 and GBM2 subtypes.

To explore the prognostic ability of LRGs in GBM, unsupervised clustering was performed using the NMF algorithm based on the expression of 17 prognostic LRGs in 168 GBM patients. The optimal number of clusters was determined to be two, resulting in the classification of samples into two distinct subtypes: GBM1 (n = 50) and GBM2 (n =118) (**Figures 2A, B**; **Supplementary Figure 2**). Principal component analysis (PCA) further confirmed that these subtypes could be effectively distinguished based on the expression profiles of the 17 LRGs (**Figure 2C**). Kaplan–Meier survival analysis revealed that patients in the GBM1 group had significantly shorter OS compared to those in the GBM2 group (*P* = 0.031) (**Figure 2D**). Expression analysis showed substantial differences in LRG expression between the two clusters (**Figure 2E**). Specifically, LCP1, LSP1, PDLIM1, PFN1, LGALS1, S100A11, and S100A4 were highly expressed in the GBM1 cluster, while SARNP, UBE2E1, RPL13, RPL5, EEF1A1, RPL29, and RPS11 were significantly upregulated in the GBM2 cluster. Additionally, among the 168 GBM patients, only 8 exhibited IDH1 missense mutations. Notably, all 8 patients belonged to the GBM2 group, consistent with GBM2 higher overall survival compared to the GBM1 group.

### TME characteristics in GBM clusters

**Figure 3:**
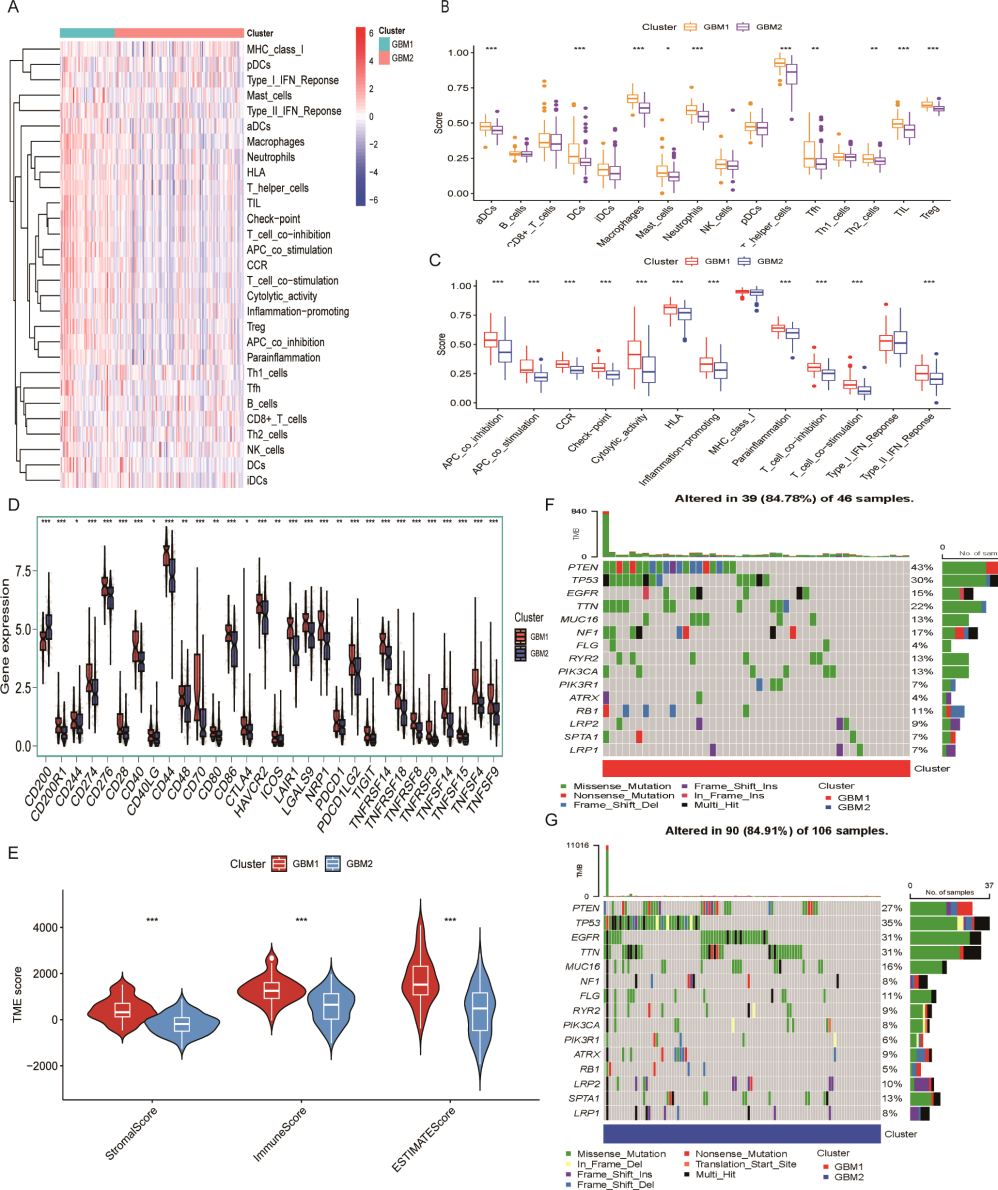
Tumor microenvironment characteristics of lactylation-based GBM subtypes. **(A)** Heatmap of 28 immune cell infiltration profiles across samples. **(B)** Comparison of 16 immune cell infiltration scores between GBM1 and GBM2 subtypes. **(C)** Comparison of 13 immune-related pathway activity scores between subtypes. **(D)** Expression levels of 30 immune checkpoint genes in the two GBM subtypes. **(E)** ESTIMATE-derived immune and stromal scores in GBM1 and GBM2. **(F)** Waterfall plot of somatic mutation frequencies in the GBM1 cluster. **(G)** Waterfall plot of somatic mutation frequencies in the GBM2 cluster. *P < 0.05; **P < 0.01; ***P < 0.001.

The TME plays a critical role in glioma progression, encompassing both tumor and non-tumor components, including innate and adaptive immune cells with pro- or anti-tumor activities. To investigate the immunological landscape of the identified GBM subtypes, we applied the ssGSEA algorithm to quantify the infiltration of 29 immune cell types in each sample (**Figure 3A**). Using the Wilcoxon rank-sum test, we compared the abundance of 16 immune cell types across two GBM clusters and found significant differences in 10 of them. These included activated dendritic cells (aDCs), DC8, macrophages, mast cells, neutrophils, T helper cells, T follicular helper (Tfh) cells, Th2 cells, tumor-infiltrating lymphocytes (TILs), and regulatory T cells (Tregs) (**Figure 3B**). Furthermore, the activity of 13 immune-related functions was analyzed between the two subtypes. Significant differences were observed in 11 immune functions (**Figure 3C**), including APC_co_inhibition, APC_co_stimulation, chemokine receptor (CCR), Check-point, Cytolytic activity, HLA, Inflammation-promoting, Para inflammation, T_cell_co-inhibition, T_cell_co-stimulation, and Type_II_IFN_Response. These findings indicate that the GBM1 subtype exhibits a more immunologically active TME, as indicated by higher immune cell infiltration and immune function scores. Consistent with this, expression analysis of 30 immune checkpoint genes revealed generally elevated levels in the GBM1 cluster (**Figure 3D**).

We further evaluated the stromal and immune scores using the ESTIMATE algorithm. Both scores were significantly higher in GBM1 than in GBM2, indicating greater infiltration of stromal and immune cells within the TME of the GBM1 subtype (**Figure 3E**). Given that tumor genomic alterations are closely associated with immune phenotypes, we examined the somatic mutation landscape of the two GBM subtypes using TCGA mutation data. While the most frequently mutated genes, including TP53, EGFR, TTN, MUC16, FLG, ATRX, LRP2, SPTA1, and LRP1, were common to both subtypes, they occurred at higher frequencies in the GBM2 group (**Figures 3F, G**). These findings highlight distinct genomic and immunological profiles between GBM1 and GBM2.

### GO and KEGG Pathway enrichment analysis

**Figure 4:**
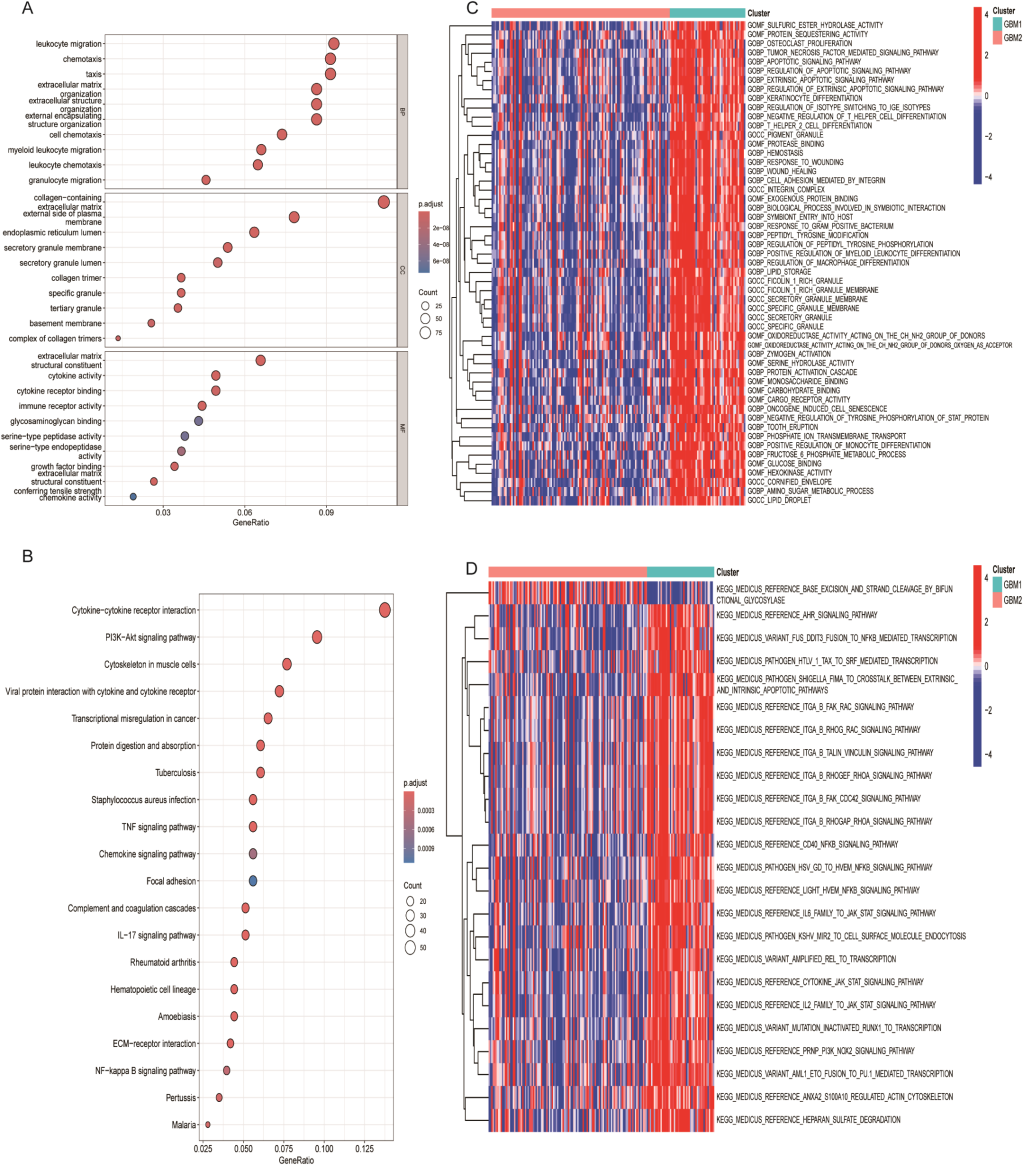
Functional enrichment analysis of lactylation-related DEGs. **(A)** Gene Ontology (GO) enrichment analysis of DEGs. **(B)** KEGG pathway enrichment analysis of DEGs. **(C)** Heatmap of enriched hallmark gene sets between GBM1 and GBM2. **(D)** Heatmap of enriched KEGG pathways distinguishing the two subtypes.

To further investigate the molecular differences between the GBM1 and GBM2 subtypes, we performed differential expression analysis. Using a significance threshold of P < 0.05 and |log_2_ FC| > 1, a total of 849 DEGs were identified between the two groups. These DEGs were subsequently subjected to GO and KEGG enrichment analyses to explore their biological implications. GO enrichment analysis revealed significant enrichment in biological processes (BP) related to the positive regulation of cytokine production and in cellular components (CC) associated with secretory granule membranes. KEGG pathway analysis showed notable enrichment in the cytokine–cytokine receptor interaction pathway (**Figures 4A, B**), suggesting active immune and inflammatory signaling differences between the two subtypes. To gain a more detailed understanding of the functional divergence, GSVA was conducted to evaluate enrichment scores of GO and KEGG terms across subtypes. In GBM1, the enriched GO terms included sulfuric ester hydrolase activity, protein sequestering activity, and osteoclast proliferation (**Figure 4C**). KEGG pathway enrichment in GBM1 highlighted pathways such as AHR signaling, and NF-κB-mediated transcription activation (**Figure 4D**), indicating subtype-specific involvement in inflammation and immune regulation.

### Drug sensitivity profiling

**Figure 5:**
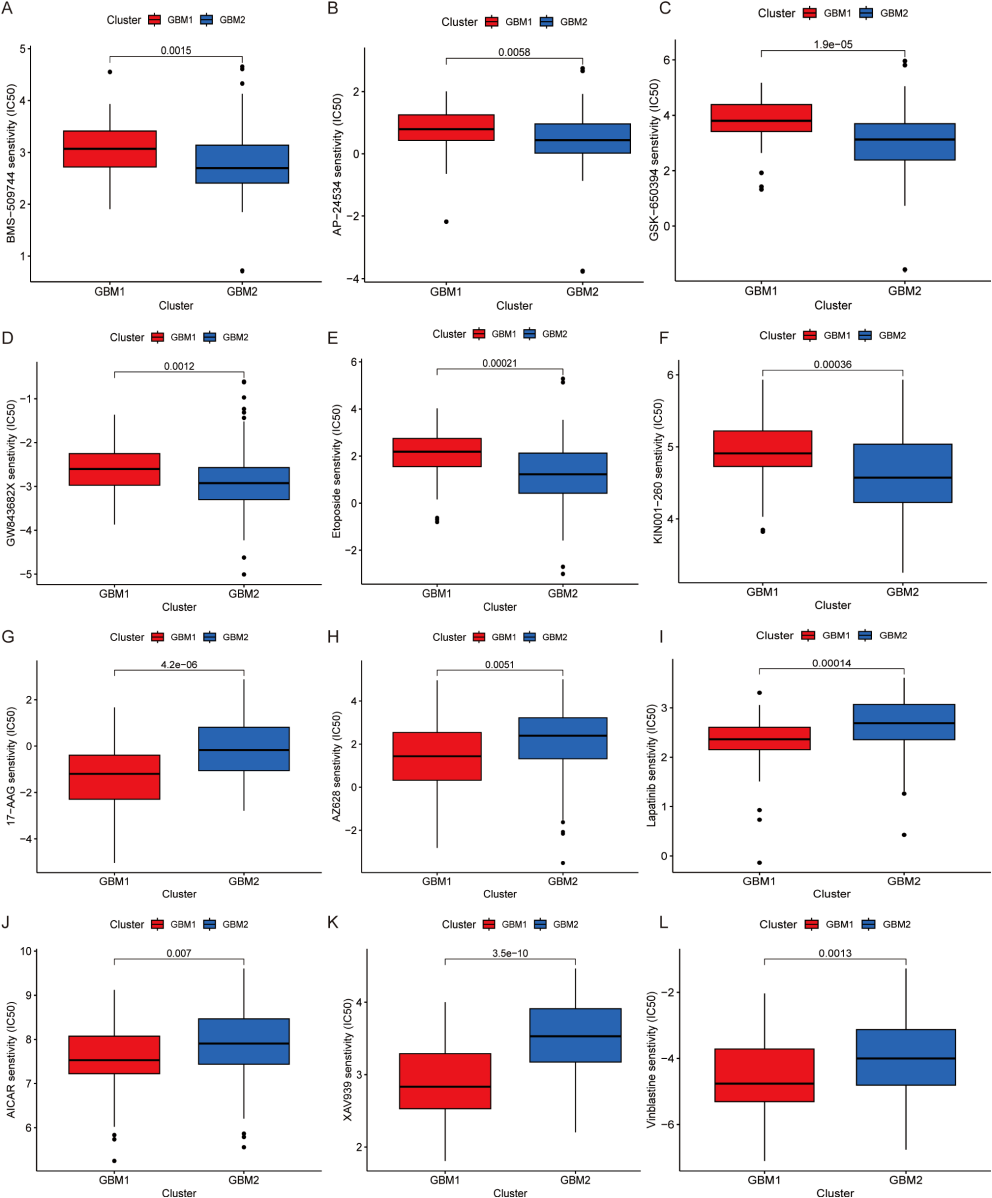
Drug sensitivity analysis of GBM subtypes. Comparison of estimated IC50 values for various drugs is illustrated. **(A)** BMS-509744, **(B)** AP-24534, **(C)** GSK-650394, **(D)** GW843682X, **(E)** Etoposide, **(F)** KIN001-260, **(G)** 17-AAG, **(H)** AZ628, **(I)** Lapatinib, **(J)** AICAR, **(K)** XAV939, **(L)** Vinblastine.

To assess the therapeutic relevance of the identified GBM subtypes, we performed drug sensitivity analysis based on gene expression data using the pRRophetic algorithm. A panel of chemotherapeutic and targeted agents was evaluated by estimating the half-maximal inhibitory concentration (IC50) values for each subtype. GBM1 exhibited significantly higher IC50 values for several compounds, including BMS-509744, AP-24534, GSK-650394, GW843682X, etoposide, and KIN001-260 (**Figures 5A–F**), suggesting that these drugs may be more effective in the GBM2 subtype. In contrast, GBM1 demonstrated greater sensitivity to 17-AAG, AZ628, lapatinib, AICAR, XAV939, and vinblastine, as indicated by lower IC50 values (**Figures 5G–L**). These findings imply subtype-specific vulnerabilities that could inform personalized treatment strategies.

### Weighted gene co-expression network analysis of GBM subtypes

**Figure 6:**
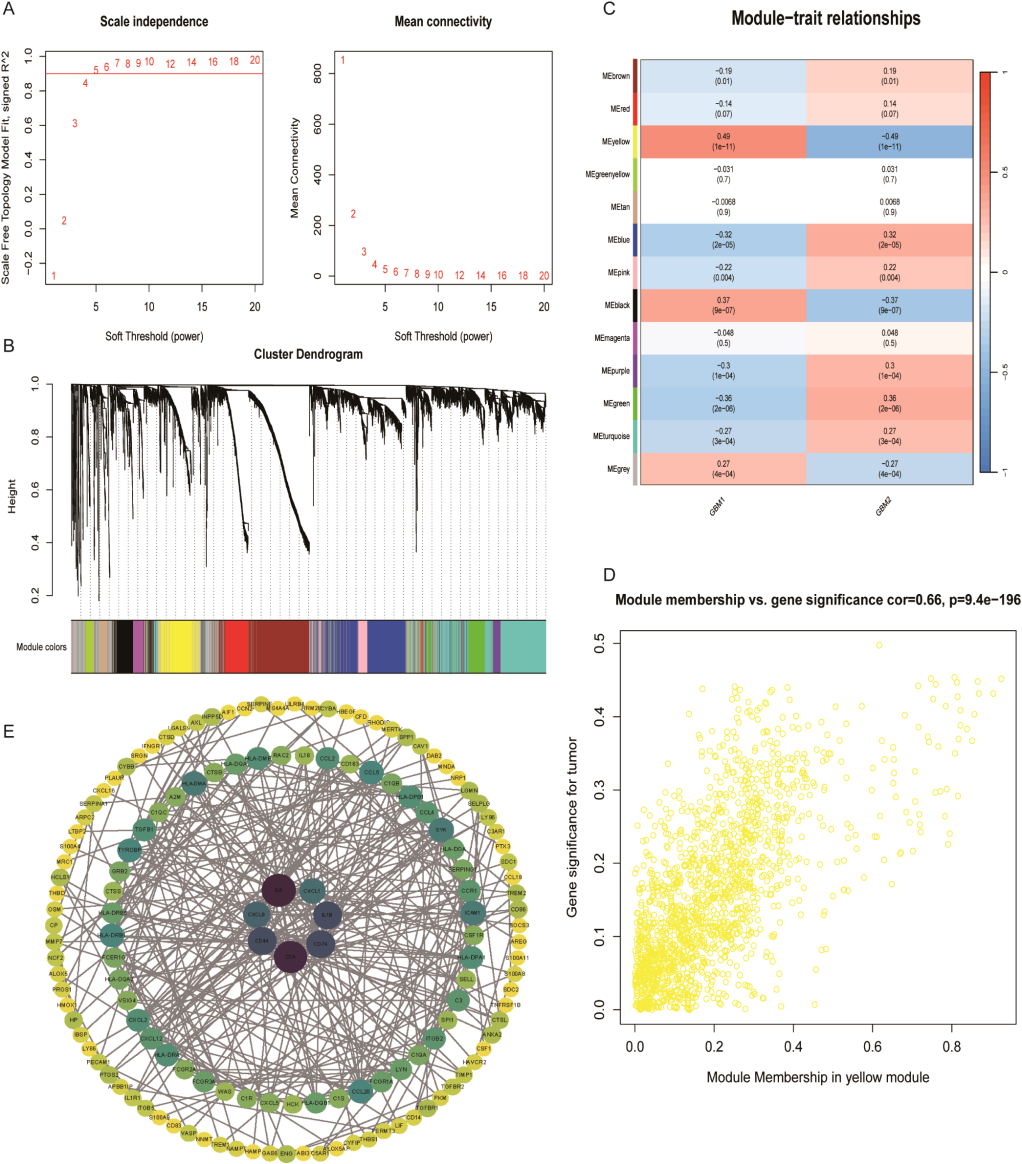
Co-expression network analysis of lactylation-related genes. **(A)** Scale-free topology model fit and soft-thresholding power selection. **(B)** Gene dendrogram after dynamic tree cutting and module merging. **(C)** Heatmap showing the correlation between 13 gene modules and GBM subtypes. **(D)** Key genes in the yellow module with correlation coefficients > 0.5. **(E)** Protein–protein interaction network of representative genes in the turquoise module.

To identify transcriptional modules driven by LRG in GBM subtypes, WGCNA was conducted. A total of 13 co-expressed gene modules were identified. Of these, three modules (yellow, black, and grey) demonstrated strong positive correlation with GBM1 and negative correlation with GBM2, whereas modules including brown, red, blue, pink, purple, green, and turquoise were predominantly associated with GBM2 (**Figure 6**). Within the yellow module, 399 hub genes were identified as central to the lactylation-related regulatory network. A PPI network constructed from this module revealed seven key hub genes: CD4, IL6, CD44, CD74, IL1B, CXCL1, and CXCL8. These genes are well-known immune and inflammatory mediators, reinforcing the immunologically active profile of the GBM1 subtype.

### Single-cell transcriptomic mapping of lactylation-associated genes

**Figure 7:**
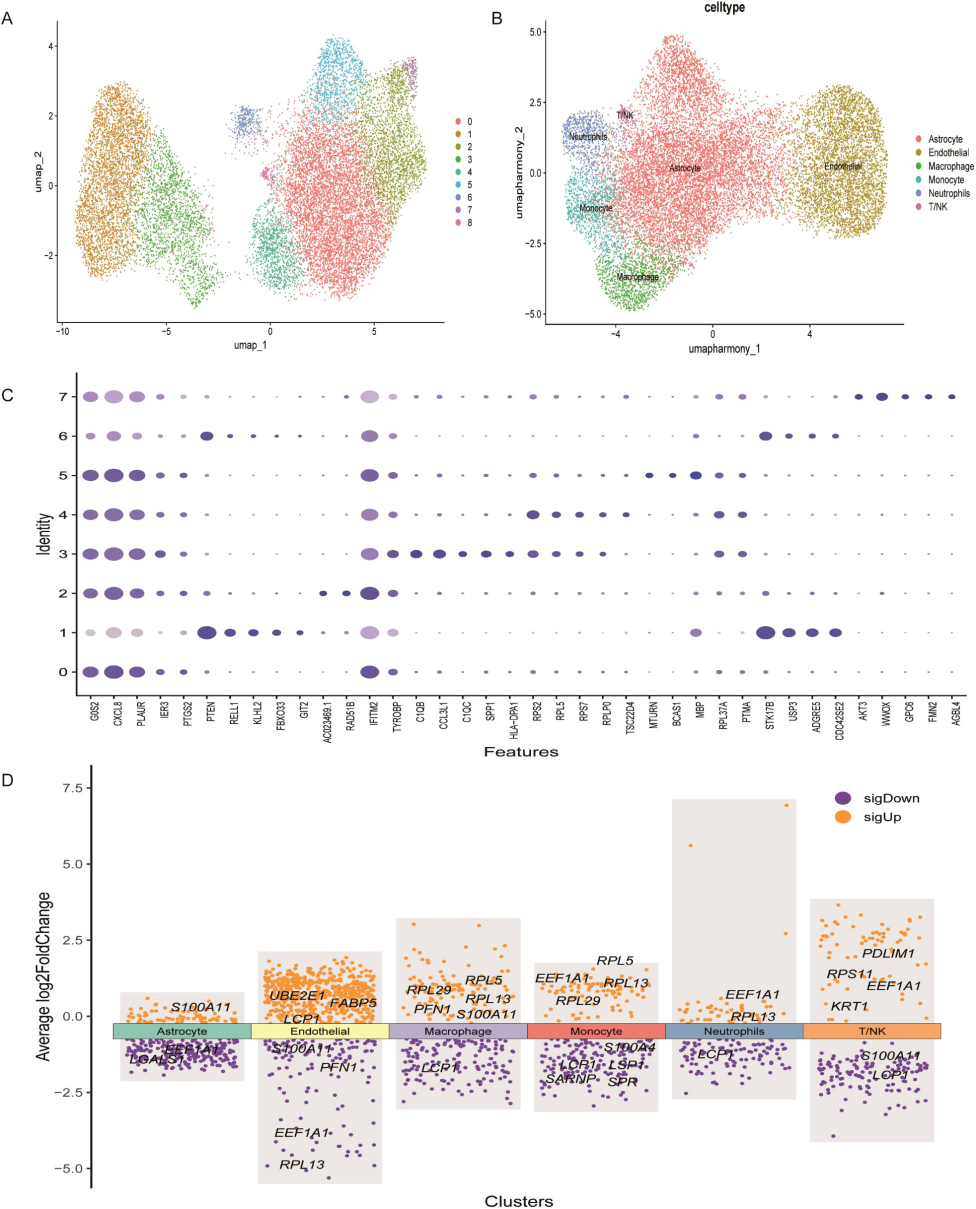
Heterogeneity in the expression of key genes in GBM at the single-cell level. **(A)** UMAP plot showing distinct cell subsets identified in GBM samples. **(B)** Annotation of cell types within GBM tissues based on single-cell RNA sequencing data. **(C)** Expression profiles of representative marker genes and lactylation-related key genes across different cellular subpopulations. **(D)** Heatmap displaying the expression levels of key lactylation-related genes across annotated cell types.

scRNA-seq data were analyzed using the Seurat package in R. Low-quality cells were excluded by filtering based on the percentage of mitochondrial and ribosomal gene expression. Data normalization was performed using the normalizedata function, and the top 2,000 highly variable genes were identified using findvariablefeatures (**Supplementary Figures 3A–D**). Batch effects between samples were corrected using the Harmony algorithm, and Uniform Manifold Approximation and Projection (UMAP) was applied for dimensionality reduction and clustering. A total of 25,000 tumor-derived cells were clustered into nine distinct groups (**Figure 7A**). Based on marker gene expression profiles (**Figure 7B**), these clusters were annotated into six main cell types: endothelial cells, T/NK cells, macrophages, monocytes, astrocytes, and neutrophils (**Figure 7C**). Using the FindAllMarkers function, the top three marker genes for each cell type were identified (**Supplementary Figure 3E**).

The expression patterns of the 17 prognostic LRGs across these six cell types were evaluated. FABP5, LCP1 and UBE2E1 were mainly expressed in endothelial cells, while KRT1, PDLIM1, and RPS11 were enriched in T/NK cells. PFN1, RPL29 and RPL5 were primarily expressed in macrophage cells, whereas S100A4, SARNP and SPRLSP1 were highly expressed in monocytes (**Figure 7D**). Moreover, S100A11 and LGALS1 were enriched in astrocytes, while EEF1A1 and RPL13 were predominantly expressed in neutrophils. These expression patterns reveal cell-type-specific roles of LRGs in the GBM microenvironment (**Figure 7D**).

### Downregulation of LCP1 inhibits cell proliferation and invasion

**Figure 8:**
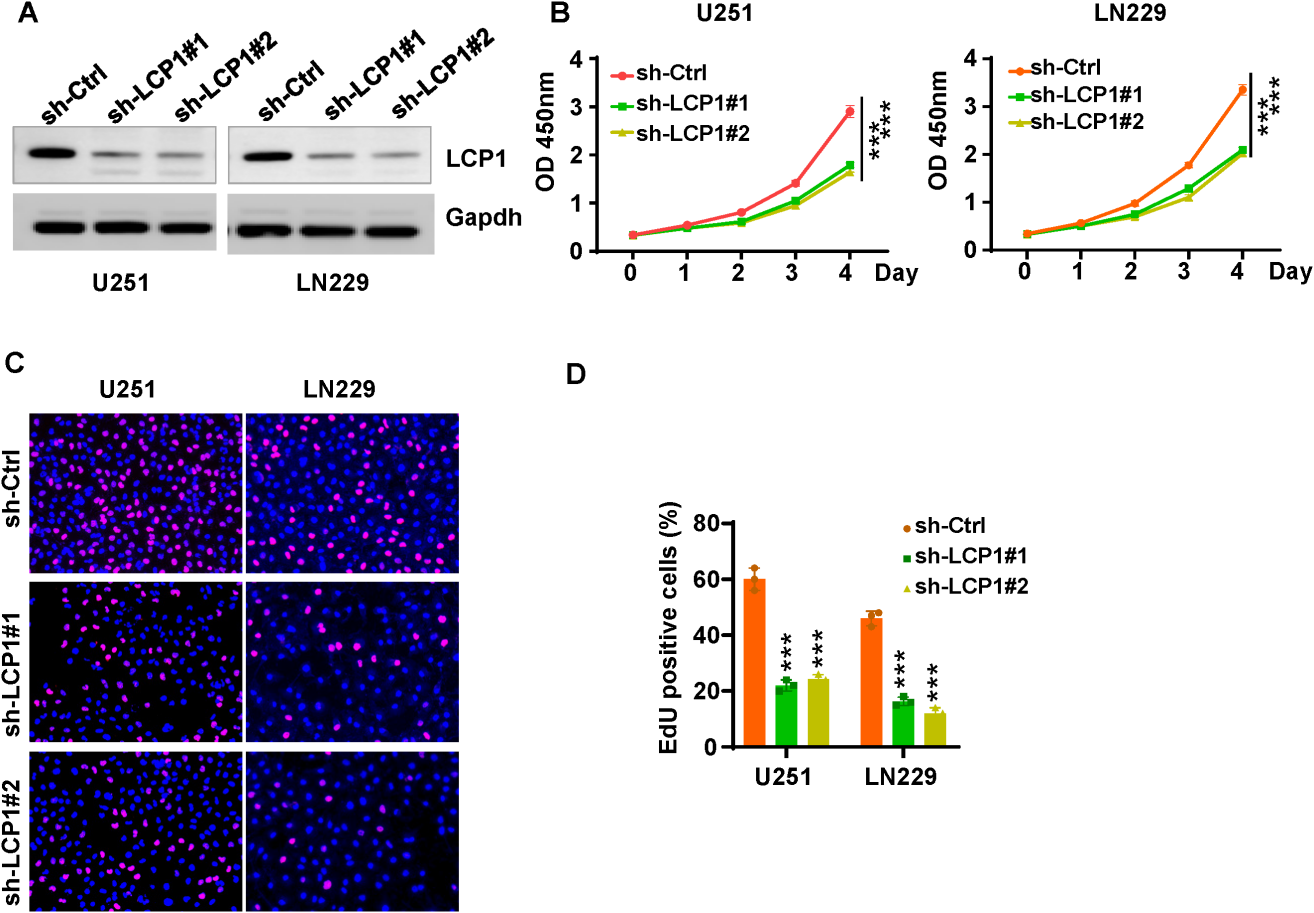
LCP1 knockdown suppresses cell viability and proliferation in glioma cells. **(A)** Western blot analysis was performed to measure the knockdown efficacy of shLCP1 in U251 and LN229 cells. **(B)** CCK-8 assays were performed to evaluate cell viability at various time points following shLCP1 transfection in U251 and LN229 cells. **(C)** EdU assays were carried out to assess cell proliferation in U251 and LN229 cells 72 hours post-transfection with shLCP1. **(D)** Quantification of EdU-positive cells in U251 and LN229 cells following LCP1 knockdown. ***p < 0.001.

**Figure 9:**
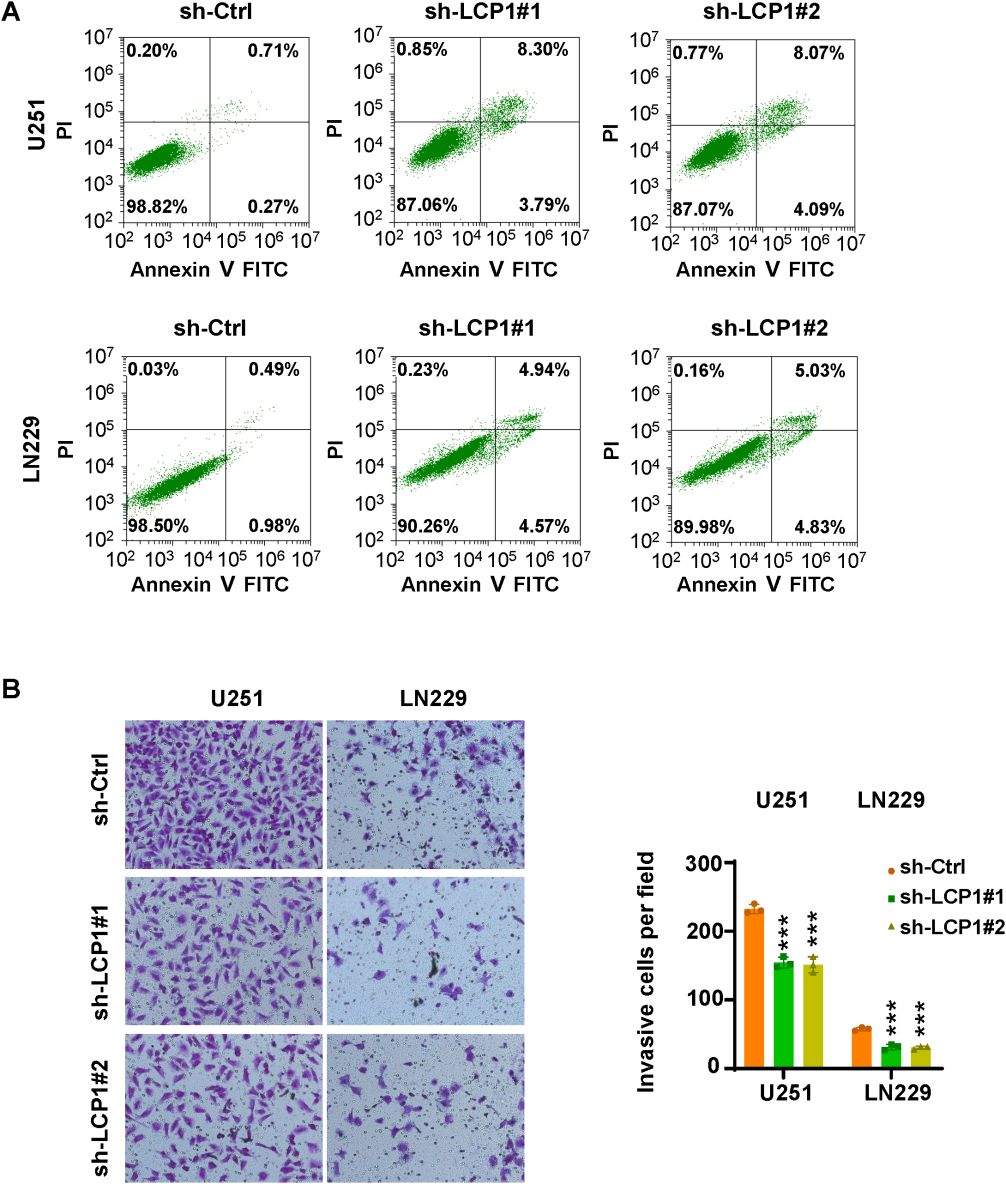
LCP1 knockdown induces apoptosis and inhibits cell invasion. **(A)** Annexin V-FITC/PI staining was used to evaluate apoptosis in U251 and LN229 cells after shLCP1 transfection for 72 hours. **(B)** Left: Transwell invasion assays were conducted to assess the invasive capacity of U251 and LN229 cells 24 hours post-transfection with shLCP1. Right: Quantification of invaded cells from the Transwell assay. ***p < 0.001..

Among 17 LGRs, LCP1, LSP1, PDLIM1, PFN1, LGALS1, S100A11, and S100A4 were highly expressed in the GBM1 cluster, while SARNP, UBE2E1, RPL13, RPL5, EEF1A1, RPL29, and RPS11 were upregulated in the GBM2 cluster. Given that patients in the GBM1 cluster had significantly shorter overall survival than those in GBM2, we prioritized the seven genes enriched in GBM1 for further investigation. Upon reviewing the literature, we found that the functions of LSP1 (29), PDLIM1 (30), PFN1 (31), LGALS1 (32), S100A11 (33), and S100A4 (34) have already been studied in the context of GBM. However, LCP1 has not been characterized in GBM, making it a novel and promising candidate for functional validation. To explore the role of LCP1 in glioma, we transfected U251 and LN229 cells with shLCP1 to knock down the expression of LCP1. We chose LN229 and U251 cells to reflect the phenotypic diversity of GBM. LN229 represents a more epithelial-like phenotype with moderate invasiveness, while U251 exhibits a mesenchymal-like phenotype and higher invasive potential. This diversity allowed us to assess whether the effects of LCP1 knockdown were consistent across distinct GBM subtypes. Western blot analysis confirmed that shLCP1 effectively reduced LCP1 protein levels in both glioma cell lines (**Figure 8A**). CCK-8 assays demonstrated that downregulation of LCP1 significantly suppressed cell viability in U251 and LN229 cells (**Figure 8B**). Similarly, EdU incorporation assays showed that downregulation of LCP1 suppressed cell proliferation in both U251 and LN229 cells (**Figures 8C, D**). Notably, Annexin V-FITC/PI staining revealed that LCP1 depletion by shLCP1 transfection induced apoptosis in U251 and LN229 cells (**Figure 9A**). To assess the effect of LCP1 on cell invasive ability, Transwell invasion assay were performed in U251 and LN229 cells, showing that LCP1 knockdown markedly reduced the invasive capacity of both glioma cell lines (**Figure 9B**). Taken together, our findings indicated that oncogenic role of LCP1 is not limited to a specific phenotypic background. These results support the robustness of LCP1 as a functional target in GBM regardless of mesenchymal or epithelial-like characteristics.

## Discussion

By integrating bulk and scRNA data with LRGs, this study comprehensively elucidates the multifaceted role of lactylation in GBM, specifically in prognostic prediction, molecular subtyping, and tumor immune microenvironment characterization, and potential therapeutic targeting (35, 36). We identified 17 lactylation-associated prognostic genes and stratified GBM patients into two distinct subtypes (GBM1 and GBM2), which exhibited significance differences in overall survival, immune cell infiltration, immune checkpoint expression, and somatic mutation profiles. This work establishes the first systematic framework linking lactylation to GBM classification, prognosis, and precision treatment strategies, offering novel insights for individualized therapeutic approaches.

Lactate, a key metabolite of the TME, not only fuels tumor energy metabolism via the Warburg effect but also regulates gene expression through lactylation-based epigenetic modifications. All 17 LRGs identified, including EEF1A1, RPL family, S100A family members, were associated with poor prognosis (HR > 1) in univariate Cox analysis, suggesting their oncogenic roles in GBM progression (37). Notably, the strong positive correlation between ribosomal proteins (such as RPL13 and RPL5) and EEF1A1 suggests that lactylation may drive the malignant phenotype of GBM by enhanced ribosome biogenesis and protein translation efficiency. Additionally, S100A11 and S100A4, as calcium-binding proteins (38, 39), have been reported to promote tumor metastasis by activating inflammatory pathways such as NF-κB (40, 41). Their co-expression with immune-related genes such as LCP1 and LGALS1 further suggests that lactylation may affect GBM progression by coordinating inflammatory response and immune evasion. The high interaction among these genes in the PPI network supports their cooperative function in functional pathways, which may jointly constitute a potential “lactylation regulatory network” driving GBM aggressiveness.

Based on the NMF-based classification of 17 prognostic LRGs, GBM patients were divided into GBM1 (high immune infiltration type) and GBM2 (low immune infiltration type). These subtypes exhibited significant differences in survival outcomes, tumor immune microenvironment characteristics, and genomic profiles. TME analysis revealed that GBM1 subtype was associated with higher immune cell infiltration, particularly macrophages and TILs, and elevated expression of immune checkpoint molecules such as PD-L1 and CTLA-4, suggesting a greater potential responsiveness to immune checkpoint inhibitors (42). On the contrary, the immunosuppressive microenvironment observed in the GBM2 subtype may be linked to its higher mutation burden (such as EGFR, TTN) and activation of ribosomal-related pathways. Previous studies have demonstrated that GBM subtypes with heightened immune infiltration are generally more responsive to immunotherapy (43, 44), while metabolic reprogramming, such as lactate accumulation, can promote immune escape by inhibiting T cell function (45, 46). Moreover, the elevated mutation frequencies of TP53 and ATRX in the GBM2 may contribute to increased genomic instability, further exacerbating prognosis (47, 48). These findings emphasize the importance of combining molecular subtyping and TME characteristics to guide individualized treatment.

GO and KEGG enrichment analyses indicated that the cytokine-receptor interaction pathway was significantly activated in the GBM1 subtype, including the IL-6/JAK/STAT3 signaling. This is consistent with the high expression of PFN1 and RPL29 in macrophages, as identified through scRNA seq results. Macrophages, as primary source of pro-inflammatory factors in the TME (49, 50), may undergo lactylation modifications that facilitate the formation of an immunosuppressive microenvironment by regulating the secretion of cytokines such as IL-6 and CXCL8 (51). Additionally, GSVA analysis demonstrated enrichment of the sulfate hydrolase activity pathway in GBM1, suggesting that lactylation may influence extracellular matrix (ECM) remodeling through sulfation modification, thereby enhancing tumor aggressiveness (52).

Drug sensitivity analysis uncovered distinct therapeutic vulnerabilities between the two subtypes. GBM2 exhibited greater sensitivity to topoisomerase inhibitors such as etoposide, potentially correlating with its higher proliferative activity. Conversely, GBM1 demonstrated increased sensitivity to 17-AAG (an HSP90 inhibitor) and lapatinib (an EGFR/HER2 inhibitor), which may be attributed to its immune cell-dependent stress response pathway (53, 54). These results offer a crucial foundation for informed clinical drug selection and personalized treatment regimens. WGCNA identified gene co-expression modules specifically associated with the GBM subtypes. In particular, the yellow module, associated with GBM1, included hub genes such as CD4, IL6, and CXCL8, which are central to immune regulation. For instance, IL6 is known to induce the polarization of M2 macrophages via STAT3 signaling (55), whereas CXCL8 enhances angiogenesis by recruiting neutrophils (56, 57). Single-cell sequencing further unveiled cell type-specific expression patterns of LRGs. For example, FABP5 and UBE2E1 were highly expressed in endothelial cells, potentially promoting angiogenesis by regulating fatty acid metabolism (58). Conversely, the enrichment of RPL5 and RPS11 in T/NK cells indicates that lactylation may influence lymphocyte function and thus modulate the anti-tumor immune response.

Given the high heterogeneity of GBM, the prognosis of IDH wild-type (WT) GBM patients is generally worse than that of IDH mutant (MUT) patients (59). One study identified two distinct immune infiltration subtypes in GBM, with Gene Cluster A characterized by a hot immune phenotype, low IDH1 mutation frequency, elevated immune-related gene expression, and favorable prognosis and immunotherapy response, whereas Cluster B exhibited a cold immune phenotype, high expression of neuronal-related genes, low immune reactivity, and lacked such clinical benefits (60). Another study identified ten ferroptosis-related genes associated with IDH1 status in GBM, which highlight their potential as biomarkers and therapeutic targets, especially in IDH1 WT GBM with poor prognosis (61). A pathway-based classification of IDH WT GBM identified four metabolically and developmentally distinct subtypes, revealing that mitochondrial GBM exhibits the best prognosis and unique vulnerability to oxidative phosphorylation inhibitors, offering new avenues for precision metabolic therapy (62). In our study, IDH1 missense mutations were found in only 8 of 168 GBM patients, all within the GBM2 subgroup with favorable survival, suggesting a potential association between IDH1 mutations and improved prognosis.

LCP1 has been reported to be involved in tumorigenesis and progression across various cancer types. For instance, one study demonstrated that LCP1 contributes to olaparib resistance by activating the JAK2/STAT3 signaling pathway in ovarian cancer (63). Additionally, LCP1 has been identified as a contributor for chidamide resistance in gastric cancer (64). Another study reported that LCP1 is associated with immune infiltration and may serve as a prognostic biomarker in triple-negative breast cancer (65). Similarly, LCP1 has been validated as a prognostic biomarker and is linked to immune infiltrate in gastric cancer (66). In the present study, we found that LCP1 promotes cell proliferation and invasion while inhibiting apoptosis in glioma cells. The consistent effects of LCP1 knockdown in both epithelial-like LN229 and mesenchymal-like U251 cells underscore its broad oncogenic role and support its potential as a promising therapeutic target across diverse GBM phenotypes.

Utilizing orthotopic GBM mouse models with LCP1 knockdown would provide *in vivo* validation of the *in vitro* findings. Moreover, no evidence has been presented to confirm whether LCP1 is directly regulated through lactylation. Besides LCP1, other 16 LRGs such as LGALS1 and RPL29 should be validated in glioma progression in future studies This study has several limitations that should be acknowledged. The sample size is small, particularly the small number of normal brain tissue samples, which may affect the robustness and accuracy of DEG identification. Although unsupervised clustering analysis was performed, the predictive capability of the model was not validated using an independent test set. As a result, the model may be overfitted to the specific patterns of the training data. In such cases, the clustering results may serve only as a descriptive analysis of the training set and may not generalize well to new or unseen data. There is a lack of *in vitro* and *in vivo* experiments to verify the specific regulatory mechanism by which lactylation influences gene expression and function. It is important to note that direct detection of protein lactylation using mass spectrometry or western blotting was not performed in this study, highlighting a key direction for future research. Absence of clinical treatment response data limits the direct clinical translational value of the molecular subtyping model. This study is constrained by its dependence on retrospective public datasets, and validation with patient-derived samples is essential to confirm the robustness of the identified subtypes. Moreover, whether lactylation-related signatures serve as diagnostic biomarkers in gliomas remains unclear, which warrants further in-depth investigation. Future studies should leverage organoid models or spatial transcriptomics to further investigate the role of lactylation in GBM cell-microenvironment interactions. Furthermore, prospective cohort studies are warranted to validate the prognostic and therapeutic power of the prognostic models.

## Conclusion

This study systematically clarified the pivotal role of LRGs in GBM prognosis, molecular classification, and immune microenvironment modulation. The identification of two molecular subtypes, GBM1 and GBM2, provides a novel framework for personalized treatment stratification in GBM. These findings highlight the clinical potential of targeting lactylation and de-lactylation pathways and underscore the need for further research into the crosstalk between lactylation modifications and the tumor immune microenvironment. Such efforts may ultimately pave the way for the development of innovative immunometabolic therapies in GBM.

## Data Availability

The original contributions presented in the study are included in the article/**Supplementary Material**. Further inquiries can be directed to the corresponding author.
