# Supplementary material for: Lactylation-related gene signatures identify glioma molecular subtypes with prognostic, immunological, and therapeutic implications
Source: Front Oncol. 2025 Jul 16;15:1613423. doi: 10.3389/fonc.2025.1613423 (PMC12307187; doi:10.3389/fonc.2025.1613423)
Supplement: Supplementary file 1 [file DataSheet1.pdf]

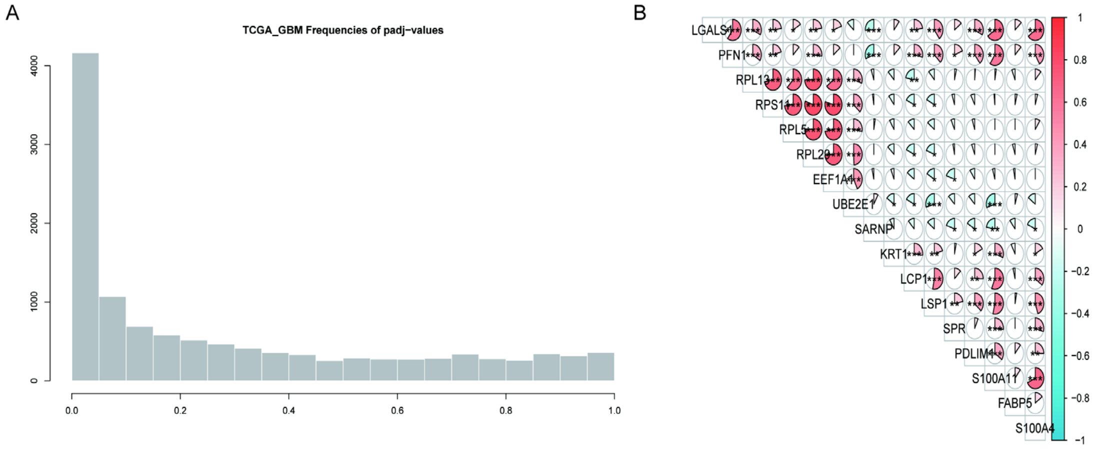

Supplementary figure 1. Overview of differentially expressed genes in GBM.

(A) Bar graph summarizing the number of upregulated and downregulated genes in TCGA_GBM samples.

(B) Correlation heatmap of differentially expressed genes associated with lactate metabolism and lactic acidosis.

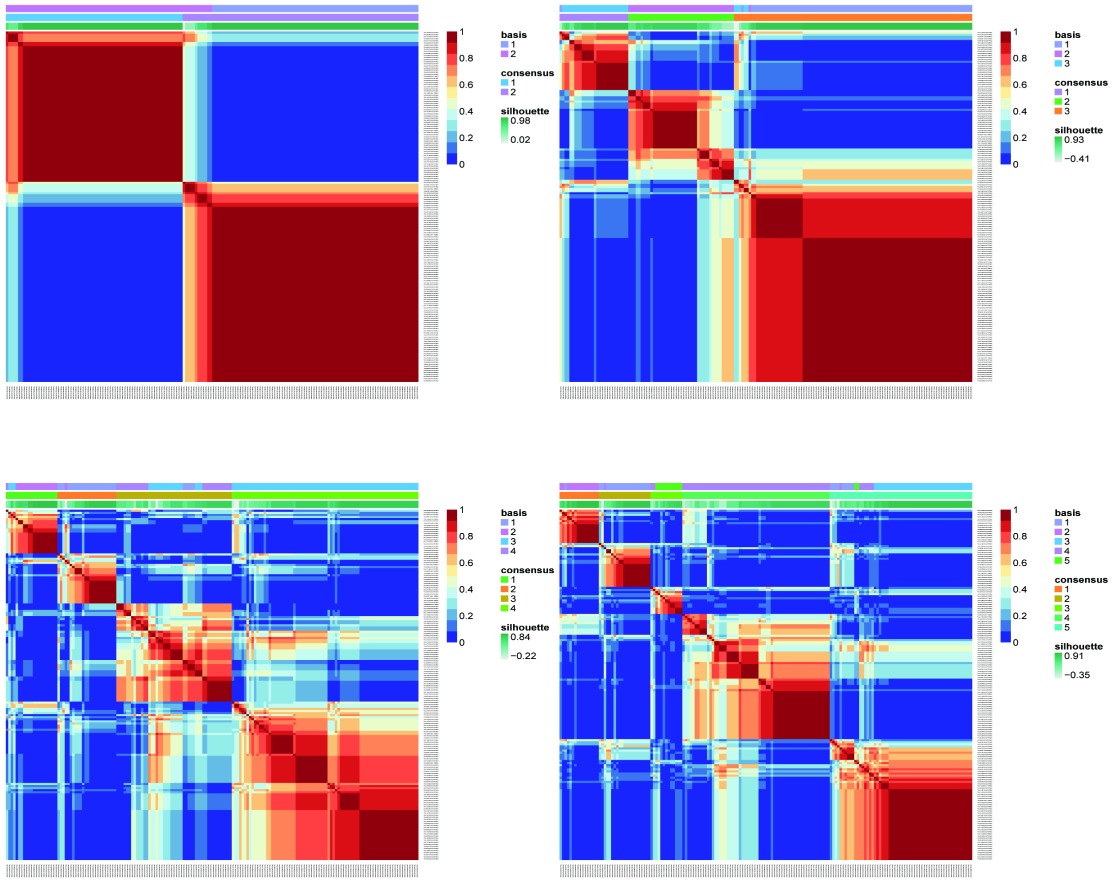

Supplementary figure 2. Unsupervised clustering analysis based on lactate-related DEGs.

NMF clustering results used to identify potential molecular subtypes of GBM based on differentially expressed genes related to lactate metabolism.

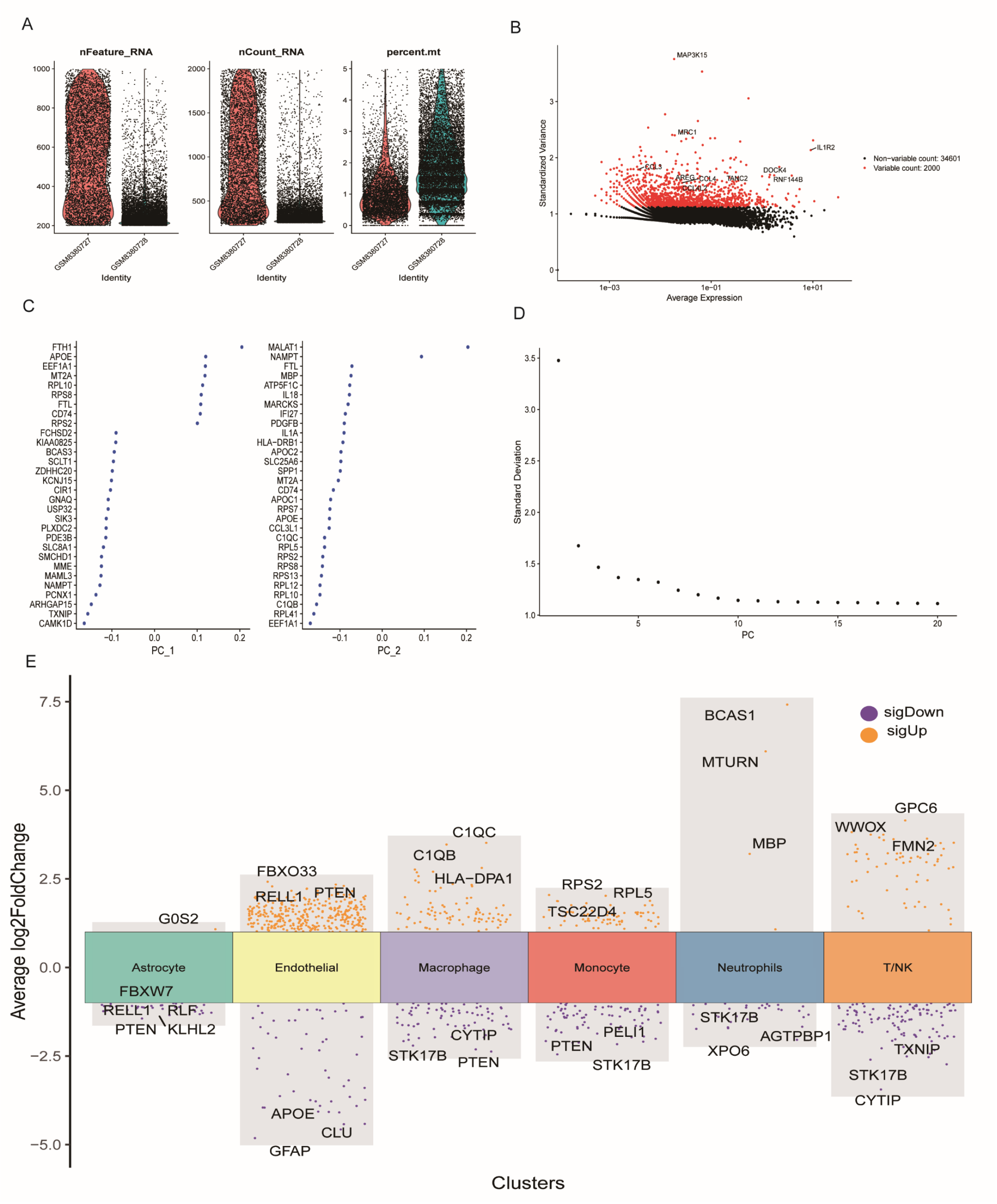

Supplementary figure 3. Single-cell transcriptomic analysis of glioma.
(A) Quality control metrics for scRNA-seq data.
(B) Volcano plot of highly variable genes identified in single-cell data.
(C) PCA plot showing linear dimensionality reduction.
(D) Elbow plot used to determine the optimal number of principal components.
(E) Volcano plot highlighting the top three highly expressed marker genes in each identified cell type.
